# Perioperative Kidney Transplant Care: A Narrative Review of Preoperative Optimization, Intraoperative Management, and Early Postoperative Allograft Surveillance

**DOI:** 10.7759/cureus.113062

**Published:** 2026-07-20

**Authors:** Fahad S Alrashidi, Raghad Alonazi, Nouf N Alazmi, Nada Alrufayyiq, Khulud Al-Humaidi

**Affiliations:** 1 Internal Medicine, Ad Diriyah Hospital, Third Health Cluster, Riyadh, SAU; 2 Medicine, Al Jouf University, Al Jouf, SAU; 3 General Medicine, Ad Diriyah Hospital, Third Health Cluster, Riyadh, SAU

**Keywords:** clinical nephrology, internal medicine, kidney transplant, preop screening, renal donor, risk assessment tools

## Abstract

Kidney transplantation is the preferred kidney replacement therapy for eligible patients with kidney failure; however, early allograft function remains vulnerable to potentially modifiable events across the perioperative continuum. Delayed graft function (DGF), hemodynamic instability, electrolyte and acid-base disturbances, vascular compromise, and surgical complications can adversely affect early recovery, prolong hospitalization, and influence subsequent graft outcomes. This narrative review summarizes evidence-based perioperative strategies designed to reduce preventable contributors to DGF and optimize early kidney allograft function, spanning recipient preparation before surgery, intraoperative anesthetic and surgical management, and structured surveillance during the immediate postoperative period.

Preoperative optimization includes assessment of cardiovascular reserve, volume status, frailty, anemia, electrolyte and acid-base abnormalities, timing of dialysis, anticoagulant and antiplatelet therapy, and immunologic risk, including crossmatch and donor-specific antibody status. Donor and graft-related determinants, particularly deceased-donor status, ischemia times, donation after circulatory death, and donor quality, should be integrated into perioperative risk stratification and monitoring intensity. During transplantation, avoidance of sustained hypotension, individualized fluid administration, use of balanced crystalloids where appropriate, early vasopressor support for vasodilatory hypotension, temperature and glucose control, and proactive management of hyperkalemia and metabolic acidosis are central to perfusion protection. Reperfusion should be treated as a critical diagnostic interval requiring prompt assessment for arterial or venous thrombosis, graft malposition, anastomotic compromise, and inadequate perfusion.

Immediate postoperative care should incorporate structured handoff, serial assessment of urine output and biochemical parameters, early Doppler ultrasonography when graft dysfunction is suspected, and timely differentiation of DGF from reversible vascular, urological, or hemodynamic complications. Enhanced Recovery After Surgery pathways may facilitate consistent multidisciplinary communication, early mobilization, analgesic optimization, and protocolized escalation. The ultimate clinical goal of this coordinated, risk-adapted perioperative strategy is to reduce preventable DGF, accelerate recognition of reversible complications, and support early allograft recovery.

## Introduction and background

Kidney transplantation is the preferred kidney replacement therapy for appropriately selected patients with kidney failure because it offers improved survival, quality of life, and freedom from long-term dialysis compared with maintenance dialysis. However, successful transplantation is not determined solely by completion of vascular and ureteric anastomoses. The perioperative period is a short but biologically consequential interval in which donor, graft, recipient, anesthetic, surgical, and postoperative factors converge to influence early allograft recovery. Although several baseline risks are non-modifiable, many perioperative exposures are potentially modifiable and may either attenuate or amplify ischemia-reperfusion injury, hemodynamic instability, and early graft dysfunction [1-3].

Kidney transplant recipients present a distinctive perioperative challenge. Most have advanced kidney failure with varying degrees of cardiovascular disease, left ventricular hypertrophy, autonomic dysfunction, vascular calcification, anemia, and reduced physiologic reserve. In addition, dialysis-dependent recipients may present for transplantation with substantial variation in intravascular volume, serum potassium concentration, bicarbonate status, and residual renal function. These factors make conventional assumptions regarding fluid responsiveness, blood pressure targets, and electrolyte tolerance less reliable than in patients with normal kidney function. Preoperative evaluation should therefore extend beyond routine surgical clearance and include assessment of cardiovascular reserve, volume status, timing of dialysis, anemia, coagulation status, medication exposure, and immunologic risk. Frailty and impaired physical performance are also clinically relevant because they may identify recipients with limited resilience to major surgery, prolonged recovery, and postoperative complications [4-6].

The perioperative risk profile is further influenced by donor and graft characteristics. Deceased-donor transplantation may involve prolonged cold ischemia time, hemodynamic instability in the donor, donation after circulatory death, or other graft-quality concerns, all of which increase susceptibility to ischemia-reperfusion injury and delayed allograft recovery. Delayed graft function (DGF), commonly defined as the requirement for dialysis within the first week after transplantation, remains one of the most important early complications following kidney transplantation. It is associated with longer hospitalization, more complex postoperative care, increased healthcare utilization, and potentially adverse long-term graft outcomes [7-11]. Therefore, donor type, anticipated cold and warm ischemia times, recipient sensitization status, crossmatch results, donor-specific antibodies, and the intended induction immunosuppression strategy should be clearly communicated among transplant surgery, anesthesia, nephrology, nursing, and postoperative care teams before incision.

DGF should not be regarded solely as an unavoidable consequence of donor or graft factors. Risk-prediction models have demonstrated that donor, recipient, and transplant-related variables can be integrated to estimate the likelihood of DGF, while contemporary surveys demonstrate substantial variation among centers in the management of DGF. In addition to its clinical consequences, DGF is associated with increased costs and longer hospital stays, emphasizing the importance of perioperative strategies that reduce avoidable graft injury and facilitate timely recognition of reversible complications [12-14].

Among potentially modifiable intraoperative exposures, hypotension is particularly important because the transplanted kidney is vulnerable to reduced perfusion during induction, vascular clamping, reperfusion, and early postoperative transfer. The newly transplanted kidney may have limited autoregulatory capacity in the setting of ischemia-reperfusion injury, vasoplegia, calcineurin inhibitor exposure, or pre-existing vascular disease. Prolonged or severe reductions in mean arterial pressure (MAP) may reduce graft blood flow and contribute to tubular injury. Perioperative anesthesia literature increasingly emphasizes perfusion-focused management through avoidance of sustained hypotension, early recognition of vasodilatory physiology, and individualized use of vasopressors and fluids rather than indiscriminate volume administration. Recent observational evidence has further strengthened the association between intraoperative hypotension and DGF, supporting blood pressure management as a clinically actionable component of kidney transplant care [15].

Fluid therapy requires similarly careful individualization. Kidney transplant recipients may require intravascular optimization to support graft perfusion; however, excessive fluid administration may precipitate pulmonary edema, impair oxygenation, increase cardiac workload, and complicate early postoperative recovery. Historically, liberal saline-based fluid administration was frequently used to support urine output and blood pressure. However, chloride-rich solutions may worsen hyperchloremic metabolic acidosis and may adversely affect renal perfusion physiology. The BEST-Fluids randomized controlled trial demonstrated that balanced crystalloid reduced the incidence of DGF compared with saline in deceased-donor kidney transplantation, supporting balanced crystalloids as a preferred perioperative fluid option in many recipients. Nevertheless, fluid selection alone is insufficient, and the overall strategy should account for cardiac function, estimated dry weight, ongoing blood loss, vasopressor requirements, and dynamic response to graft reperfusion [16].

Metabolic and hematologic factors are also central to early allograft protection. Hyperkalemia, metabolic acidosis, hypocalcemia, hypomagnesemia, hypothermia, hyperglycemia, and anemia may destabilize the recipient during surgery and compromise myocardial performance or vascular tone at the time of graft reperfusion. These abnormalities may arise from pre-existing kidney failure, recent dialysis, transfusion, acid load, prolonged surgery, or limited clearance before graft function is established. Accordingly, perioperative care should include proactive planning for potassium control, acid-base monitoring, temperature preservation, glucose management, transfusion thresholds, and postoperative dialysis readiness when clinically indicated. Avoidance of unnecessary blood transfusion is also desirable when feasible because of its potential implications for sensitization and subsequent immunologic complexity.

Reperfusion is a pivotal intraoperative event and should be approached as both a therapeutic and diagnostic interval. Adequate graft color, turgor, arterial pulsatility, venous drainage, and urine production may provide early clues regarding allograft perfusion, but none should be interpreted in isolation. Poor graft appearance, absent perfusion signals, or persistent oliguria should prompt simultaneous reassessment of recipient hemodynamics and potential technical causes, including arterial or venous thrombosis, kinking, torsion, anastomotic compromise, graft malposition, or external compression. Delays in identifying these complications may convert potentially reversible events into irreversible graft injury. Therefore, a low threshold for direct surgical reassessment and intraoperative Doppler evaluation, where available, is appropriate when perfusion is uncertain.

The immediate postoperative period remains equally important because DGF, hemodynamic injury, vascular compromise, urinary obstruction, and acute rejection may initially present with overlapping clinical features, including oliguria, rising serum creatinine, hyperkalemia, fluid overload, and persistent acidosis. A structured postoperative handoff should communicate donor and recipient risk factors, ischemia times, intraoperative blood pressure exposure, fluid balance, urine output, transfusion requirements, induction immunosuppression, anticoagulation plans, and specific triggers for urgent graft Doppler ultrasonography or surgical review. Early surveillance should prioritize timely differentiation between expected ischemia-reperfusion-related delayed recovery and reversible vascular or urological complications.

Enhanced Recovery After Surgery (ERAS) pathways provide a practical framework for converting these principles into reproducible multidisciplinary care. Kidney transplant ERAS recommendations emphasize coordinated preoperative preparation, multimodal analgesia, early mobilization and feeding, standardized communication, and protocolized escalation pathways. Available implementation studies suggest that ERAS pathways are feasible in kidney transplantation and may improve recovery-related outcomes while maintaining attention to graft-specific complications, including DGF [17-20]. ERAS should not be considered a rigid one-size-fits-all protocol; rather, it should serve as an implementation structure that standardizes essential processes while allowing hemodynamic targets, fluid therapy, monitoring intensity, and postoperative interventions to be adapted to recipient and graft risk.

This narrative review examines the evidence-based determinants of early allograft function across the perioperative continuum. It integrates preoperative recipient and graft risk assessment, intraoperative anesthetic, hemodynamic, metabolic, and surgical management, and immediate postoperative surveillance for DGF and reversible graft complications. The objective is to provide a clinically practical framework that supports perfusion protection, early recognition of time-sensitive complications, and consistent multidisciplinary decision-making during the period in which early allograft outcomes are most vulnerable.

## Review

Methods

This article is a narrative, practice-oriented review of perioperative kidney transplant care and early allograft function. A structured literature search was performed to identify clinically relevant evidence addressing preoperative recipient optimization, donor and graft risk factors, intraoperative anesthetic and surgical management, DGF, fluid and hemodynamic strategies, reperfusion assessment, and immediate postoperative allograft surveillance. The databases searched included PubMed/MEDLINE, Google Scholar, Scopus, and the Cochrane Library. The search covered literature from database inception through July 2026.

Search terms included combinations of the following keywords and Medical Subject Heading (MeSH)-related terms: “kidney transplantation,” “renal transplantation,” “perioperative care,” “intraoperative management,” “delayed graft function,” “early allograft function,” “ischemia-reperfusion injury,” “intraoperative hypotension,” “mean arterial pressure,” “balanced crystalloids,” “saline,” “BEST-Fluids,” “fluid therapy,” “vasopressors,” “reperfusion,” “Doppler ultrasound,” “vascular complications,” “urological complications,” “Enhanced Recovery After Surgery,” and “ERAS.” Boolean operators were used where appropriate, including combinations such as “kidney transplantation AND delayed graft function,” “kidney transplantation AND intraoperative hypotension,” “kidney transplantation AND balanced crystalloids,” and “kidney transplantation AND ERAS.”

Eligible sources included randomized controlled trials, cohort studies, clinical practice guidelines, major observational studies, systematic or narrative reviews, and clinically relevant implementation studies related to perioperative kidney transplantation and early graft outcomes. Priority was given to studies with direct relevance to perioperative decision-making, graft perfusion, DGF, postoperative surveillance, and multidisciplinary care pathways. Foundational older studies were included when they provided important definitions, risk models, or long-term outcome data, while more recent literature was prioritized for contemporary perioperative practice.

Articles were excluded if they focused exclusively on long-term outpatient transplant follow-up, chronic allograft dysfunction without perioperative relevance, pediatric transplantation only, non-kidney solid organ transplantation, experimental animal models without direct clinical applicability, or highly specialized immunologic or pharmacologic topics outside the scope of perioperative care. Because the objective was to provide a clinically applicable synthesis rather than answer a narrowly defined intervention question, no formal systematic review protocol, PRISMA flow diagram, or meta-analysis was performed. Findings were synthesized qualitatively and organized around practical perioperative phases: preoperative optimization, intraoperative management, reperfusion assessment, immediate postoperative surveillance, and ERAS-informed implementation.

Review

Preoperative Recipient Optimization and Transplant-Specific Risk Stratification

Perioperative kidney transplant care begins before the recipient enters the operating room. The preoperative objective is not simply to determine whether surgery can proceed, but to identify correctable physiologic abnormalities, define the recipient’s baseline risk, and communicate the information needed to interpret early graft function after implantation. Kidney transplant recipients frequently have advanced cardiovascular disease, left ventricular hypertrophy, autonomic dysfunction, pulmonary hypertension, vascular calcification, anemia, diabetes, and limited physiologic reserve. These conditions increase susceptibility to hypotension, pulmonary edema, perioperative ischemia, arrhythmia, and delayed recovery after surgery [1-3].

Assessment should include recent cardiovascular status, exercise tolerance or functional capacity, volume status, dialysis prescription, dry-weight trajectory, serum potassium, bicarbonate concentration, hemoglobin, platelet count, coagulation profile when indicated, and medication exposure. Frailty and impaired physical performance should also be recognized as clinically meaningful markers of reduced resilience. Frailty is not a contraindication to transplantation in isolation, but it may identify recipients at greater risk of prolonged recovery, hospitalization, and postoperative complications, thereby supporting more intensive prehabilitation, anesthetic planning, and postoperative monitoring [4-6]. 

The timing and goals of the final dialysis session require individualized planning. The recipient should ideally proceed to transplantation without clinically significant hyperkalemia, severe metabolic acidosis, uncontrolled volume overload, or symptomatic uremic complications. At the same time, excessive ultrafiltration or dialysis-associated hypotension may leave the recipient intravascularly depleted and vulnerable to induction-related hypotension. Therefore, dialysis before transplantation should aim for approximate euvolemia and biochemical optimization rather than the pursuit of a rigid weight target. Communication between nephrology, anesthesia, and surgery is particularly important when the transplant occurs soon after dialysis, when the recipient has residual pulmonary congestion, or when baseline blood pressure is chronically low.

Medication reconciliation should address antihypertensive treatment, insulin and other glucose-lowering therapies, antiplatelet agents, anticoagulants, and medications that may affect potassium balance or hemodynamic stability. The perioperative anticoagulation plan should be explicit before incision, particularly in recipients with atrial fibrillation, mechanical valves, recent venous thromboembolism, thrombophilia, antiphospholipid syndrome, prior graft thrombosis, or recent coronary intervention. A poorly coordinated anticoagulation strategy may increase either bleeding risk or the likelihood of perioperative thrombotic events.

Immunologic and transplant-specific information must also be integrated into the preoperative plan. ABO compatibility, crossmatch status, donor-specific antibodies, calculated panel-reactive antibody level, prior transplant history, intended induction therapy, and anticipated maintenance immunosuppression should be available to all core team members. These factors influence the threshold for postoperative biopsy, the interpretation of poor early graft function, transfusion avoidance strategies, and the anticipated intensity of monitoring [11]. A standardized transplant briefing should therefore include both conventional anesthetic information and transplant-critical variables, including donor type, cold and warm ischemia times, immunologic risk, last dialysis, anticoagulation status, and planned induction immunosuppression.

Donor and Graft Characteristics: Defining the Baseline Risk of Delayed Recovery

The early postoperative course is shaped by donor and graft factors that are largely established before implantation. Living-donor kidneys generally experience shorter ischemia times and lower rates of DGF than deceased-donor kidneys. In contrast, kidneys from donors after circulatory death, older donors, donors with acute kidney injury, and kidneys exposed to prolonged cold ischemia are more vulnerable to ischemia-reperfusion injury and delayed functional recovery. These factors do not mandate poor outcomes, but they should influence the anticipated probability of DGF, the level of intraoperative monitoring, the threshold for postoperative imaging, and the timing of nephrology and surgical reassessment [7-12].

Risk-prediction models for DGF have shown that recipient characteristics, donor quality, ischemia times, and transplant-related variables can be integrated to estimate the likelihood of delayed recovery [12]. Such estimates are useful when they inform communication and surveillance rather than when they are interpreted deterministically. For example, a recipient receiving a kidney with prolonged cold ischemia time may be expected to have delayed creatinine improvement; however, this expectation should never prevent urgent evaluation for vascular compromise, obstruction, or hemodynamic injury when oliguria or allograft dysfunction occurs.

Machine perfusion is increasingly relevant to perioperative kidney transplant risk assessment. Compared with static cold storage, hypothermic machine perfusion may reduce cold ischemic injury and has been associated with lower rates of DGF in selected deceased-donor kidneys. Beyond preservation, machine perfusion can provide functional and viability-related information, including flow parameters and vascular resistance trends, which may help contextualize the expected early graft recovery pattern. This is particularly important for kidneys from donors after circulatory death, older donors, or grafts exposed to prolonged ischemia. However, machine perfusion should not create false reassurance. Even when machine perfusion parameters are favorable, early postoperative oliguria or graft dysfunction still requires systematic evaluation for hemodynamic, vascular, urological, immunologic, and drug-related causes. Key perioperative risk factors, their proposed mechanisms, and practical clinical implications are summarized in Table 1.

**Table 1:** Key perioperative risk factors for early allograft dysfunction DGF, delayed graft function.

Risk factor	Mechanism	Clinical implication
Prolonged cold ischemia time	Greater ischemia-reperfusion injury and tubular injury	Higher DGF risk; closer early monitoring
Donation after circulatory death	Warm ischemic injury before organ recovery	Anticipate delayed recovery; avoid premature attribution without excluding vascular causes
Intraoperative hypotension	Reduced graft perfusion during vulnerable reperfusion period	Minimize duration and severity of hypotension
Excessive saline exposure	Hyperchloremic acidosis and possible renal perfusion effects	Prefer balanced crystalloids when appropriate
Fluid overload	Pulmonary edema, impaired oxygenation, cardiac strain	Individualize volume; avoid urine-output-driven over-resuscitation
Hyperkalemia/acidosis	Arrhythmia risk and hemodynamic instability	Plan monitoring and rapid correction; dialysis readiness
Technical vascular compromise	Thrombosis, kinking, torsion, or anastomotic obstruction	Low threshold for Doppler, surgical reassessment, and revision
Calcineurin inhibitor exposure	Afferent arteriolar vasoconstriction and functional nephrotoxicity	Individualize tacrolimus timing/target in high-DGF-risk patients
Poor handoff	Delayed recognition of reversible complications	Use structured transplant-specific communication

Transplant-Specific Handoff and Information Continuity

A transplant-specific handoff is a safety-critical intervention because omissions may delay the recognition of reversible causes of graft dysfunction. Generic surgical handoffs may document procedure type, blood loss, fluids, and anesthetic events, but kidney transplantation requires additional information that directly affects postoperative decisions. These include donor source, ischemia times, graft laterality and vascular anatomy, induction immunosuppression, crossmatch and donor-specific antibody status, intraoperative blood pressure exposure, vasopressor requirements, urine output after reperfusion, transfusion, anticoagulation, technical concerns during implantation, and any uncertainty regarding perfusion at the end of surgery [1-3].

The handoff should be structured around a shared clinical question: “What are the most plausible causes of poor early graft function in this specific recipient?” In a low-risk living-donor transplant with immediate urine production and stable hemodynamics, the initial focus may be routine surveillance. In a high-risk deceased-donor transplant with prolonged ischemia, intraoperative hypotension, vasopressor dependence, or equivocal graft reperfusion, the same postoperative findings should generate a lower threshold for urgent Doppler ultrasonography and surgical review. This approach reduces the risk that early graft dysfunction is prematurely labeled as DGF when a correctable vascular or urological complication is present.

Intraoperative Monitoring, Anesthetic Management, and Perfusion Protection

Kidney allograft perfusion depends on the interaction between recipient systemic hemodynamics, intravascular volume, vascular tone, cardiac output, surgical vascular anastomoses, and the graft’s capacity to recover after ischemia. Intraoperative monitoring should therefore be selected according to recipient and graft risk. Continuous arterial blood pressure monitoring is particularly useful in recipients with substantial cardiovascular disease, labile blood pressure, expected major fluid shifts, difficult vascular access, high DGF risk, or concern for hemodynamic instability at reperfusion [1-3].

No single MAP threshold is universally appropriate for all transplant recipients. Baseline blood pressure, chronic hypertension, recipient vascular disease, cardiac function, vasopressor exposure, and the duration of hypotension all influence the relationship between measured MAP and effective allograft perfusion. Nevertheless, the available observational data support avoiding sustained or repeated hypotension, particularly during induction, vascular clamping, reperfusion, and early emergence from anesthesia [15]. The clinical emphasis should therefore be on minimizing both the severity and duration of hypotension rather than attempting to achieve a uniform MAP target in every recipient.

When hypotension develops, management should distinguish likely hypovolemia from vasodilation, impaired cardiac output, hemorrhage, arrhythmia, or an evolving surgical problem. Repeated empiric fluid boluses may be appropriate in selected patients with true intravascular depletion, but they may be harmful in recipients with impaired cardiac reserve, pulmonary congestion, or end-stage renal disease (ESRD)-associated volume intolerance. When vasodilation is the principal mechanism, early vasopressor support may restore perfusion pressure more safely than large-volume crystalloid administration. Similarly, new hemodynamic instability after reperfusion should prompt simultaneous evaluation of recipient physiology and graft-related surgical causes rather than a purely anesthetic response.

General anesthetic management should also preserve conditions that support perfusion and postoperative recovery. Avoidance of hypothermia, severe anemia, hypoxemia, hypercapnia, and marked hyperglycemia is important because these factors can adversely affect myocardial performance, vascular tone, oxygen delivery, and wound healing. Ventilatory settings should maintain adequate oxygenation while avoiding excessive intrathoracic pressure that may impair venous return. Glucose monitoring is particularly relevant in recipients with diabetes, those receiving corticosteroid induction, and patients exposed to significant surgical stress.

Fluid Therapy: Balanced Crystalloids, Volume Individualization, and Avoidance of Chloride Burden

Fluid therapy in kidney transplantation should be directed toward adequate perfusion without precipitating pulmonary edema, right-sided cardiac strain, impaired oxygenation, and unnecessary hemodilution. Historically, liberal fluid administration and saline-based resuscitation were commonly used to support urine output after reperfusion. However, urine output alone is an imperfect surrogate for adequate allograft perfusion, particularly in the setting of osmotic diuresis, diuretic exposure, or early tubular dysfunction. Excessive fluid administration may produce transient urine output while worsening postoperative cardiopulmonary recovery.

The choice of crystalloid is clinically important. Saline contains supraphysiologic chloride concentrations and may promote hyperchloremic metabolic acidosis, which can complicate interpretation of perioperative acid-base status and may adversely affect renal perfusion physiology. In the BEST-Fluids randomized controlled trial, balanced crystalloid solution with Plasma-Lyte 148 reduced DGF compared with saline in deceased-donor kidney transplant recipients [16]. This provides the strongest available evidence supporting balanced crystalloids as the preferred routine fluid choice for deceased-donor kidney transplantation, unless a specific clinical reason favors an alternative solution.

Balanced crystalloid use should not be avoided solely because these solutions contain small concentrations of potassium. In BEST-Fluids, balanced crystalloid did not increase clinically significant hyperkalemia or serious adverse events compared with saline [16]. Hyperkalemia in kidney transplantation is more strongly influenced by recipient baseline potassium, the timing and adequacy of dialysis, metabolic acidosis, transfusion, tissue injury, and poor initial graft clearance than by the modest potassium content of balanced crystalloid solutions.

Fluid volume should remain individualized. Recipients with normal cardiac function and clear evidence of volume depletion may tolerate more generous replacement, whereas those with heart failure, pulmonary hypertension, marked left ventricular hypertrophy, or baseline pulmonary congestion require a more cautious approach. Dynamic hemodynamic assessment, serial blood pressure trends, blood loss, urine output, oxygenation, ventilatory pressures, and bedside cardiac evaluation when available are more informative than rigid volume targets. Central venous pressure should be interpreted as a contextual measurement rather than as a stand-alone endpoint for fluid administration.

Electrolyte, Acid-Base, and Metabolic Management

Hyperkalemia and metabolic acidosis are predictable perioperative hazards in recipients with kidney failure. The risk may be amplified by incomplete preoperative dialysis, prolonged fasting, insulin deficiency, tissue injury, acidosis, blood transfusion, or delayed allograft function. A preoperative potassium concentration and acid-base assessment should therefore be available before induction whenever clinically feasible. Repeat testing should be considered in prolonged procedures, before or shortly after reperfusion in high-risk recipients, after substantial transfusion, and whenever arrhythmia or hemodynamic instability occurs [1-3].

Hyperkalemia should be treated according to severity, electrocardiographic changes, and clinical urgency. Immediate stabilization of myocardial excitability, intracellular potassium shifting, correction of contributory acidosis, and definitive potassium removal when needed should be planned in advance. Postoperative dialysis should remain available for standard indications, including refractory hyperkalemia, severe metabolic acidosis, uncontrolled volume overload, and clinically significant uremic complications. The need for dialysis in the first postoperative week may meet the conventional definition of DGF, but dialysis should not be withheld when medically indicated simply to avoid classifying the recipient as having DGF.

Acid-base abnormalities require interpretation in the clinical context. Hyperchloremic metabolic acidosis may occur after saline administration, whereas lactic acidosis may suggest hypoperfusion, severe anemia, sepsis, or other systemic illness. A falling bicarbonate concentration after reperfusion should prompt assessment of fluid composition, hemodynamics, ventilation, perfusion, and possible dialysis requirements rather than reflexive bicarbonate administration alone. Magnesium, calcium, phosphate, and glucose should also be monitored because significant abnormalities may contribute to arrhythmia, reduced myocardial performance, insulin resistance, or neuromuscular dysfunction.

Hemostasis, Transfusion, and Anticoagulation Planning

Bleeding and thrombosis are competing perioperative risks in kidney transplantation. Bleeding may arise from anticoagulant exposure, uremic platelet dysfunction, extensive iliac vessel dissection, vascular calcification, or surgical technical complexity. Conversely, allograft thrombosis can result in catastrophic graft loss and may be more likely in recipients with thrombophilia, prior thrombotic events, antiphospholipid antibodies, severe atherosclerosis, or technically challenging vascular anastomoses.

The operative plan should explicitly define anticoagulant interruption, bridging where indicated, anticipated heparin use, reversal strategies, and postoperative thromboprophylaxis. Blood transfusion should be guided by active bleeding, hemodynamic status, hemoglobin trajectory, myocardial ischemia risk, and oxygen-delivery concerns rather than a fixed threshold alone. Avoidable transfusion should be minimized, particularly in sensitized recipients or those likely to require future transplantation, because transfusion may increase immunologic complexity through human leukocyte antigen (HLA) sensitization [19]. However, concern regarding sensitization should never delay transfusion when it is required to control hemorrhagic shock or maintain adequate oxygen delivery.

Reperfusion as a Therapeutic and Diagnostic Event

Reperfusion is the most critical intraoperative transition in kidney transplantation. It represents the moment at which the recipient’s hemodynamic status, graft preservation injury, vascular anastomotic integrity, and surgical positioning become clinically apparent. Adequate graft color, turgor, arterial pulsatility, venous drainage, and early urine production may be reassuring, but none alone proves sustained graft viability. Conversely, delayed urine output does not necessarily indicate technical failure, particularly in deceased-donor kidneys with substantial ischemia-reperfusion injury.

Poor graft appearance, weak arterial pulsatility, absent or patchy cortical perfusion, progressive graft congestion, unexplained hypotension, or persistent oliguria should prompt immediate parallel assessment. Recipient causes include hypotension, vasoplegia, hypovolemia, low cardiac output, hypoxemia, severe anemia, and arrhythmia. Technical causes include renal artery thrombosis, renal vein thrombosis, arterial kinking, venous outflow obstruction, torsion, graft malposition, anastomotic narrowing, external compression, and tension on the vascular pedicle. Delays in differentiating these possibilities may convert a reversible mechanical problem into irreversible allograft injury.

Intraoperative Doppler ultrasonography can provide useful information when perfusion is uncertain, particularly by assessing arterial and venous patency and identifying gross abnormalities in graft flow [20]. It should complement, rather than replace, direct surgical assessment. When vascular compromise is strongly suspected, immediate exploration and revision may be more appropriate than prolonged observation or repeated medical resuscitation. The key principle is that reperfusion abnormalities should be treated as time-sensitive diagnostic emergencies.

Immediate Postoperative Surveillance and the Differential Diagnosis of Poor Early Graft Function

The first 24 to 72 hours after kidney transplantation require structured surveillance because DGF, hemodynamic injury, vascular compromise, ureteric obstruction, urine leak, and acute rejection may initially present with overlapping features. Oliguria, rising creatinine, persistent hyperkalemia, worsening metabolic acidosis, fluid overload, graft pain, and unexplained hemodynamic instability should not automatically be attributed to ischemia-reperfusion injury.

A practical early assessment begins with confirmation of systemic hemodynamics, intravascular volume status, oxygenation, urinary catheter patency, and the presence of ongoing bleeding. Serial creatinine, potassium, bicarbonate, hemoglobin, lactate when appropriate, and urine output should be interpreted as trends rather than isolated values. Early allograft Doppler ultrasonography is appropriate when there is concern regarding arterial or venous patency, abrupt oliguria, unexplained graft dysfunction, graft tenderness, new bruit, or clinical suspicion of obstruction or external compression [20].

Vascular complications require urgent recognition because renal artery or vein thrombosis may lead rapidly to graft loss. Urological complications, including obstruction, ureteric ischemia, urinary leak, lymphocele-related compression, and perinephric collections, should also be considered, especially when hydronephrosis, decreasing urine output, rising drain output, pelvic pain, or unexplained creatinine elevation is present [18]. The evaluation should be multidisciplinary and include transplant surgery, nephrology, radiology, and anesthesia or critical care teams when systemic instability persists.

DGF is usually managed supportively after urgent vascular and urological causes have been excluded. Supportive care includes careful volume assessment, avoidance of nephrotoxins, correction of electrolyte and acid-base abnormalities, adjustment of medication dosing, dialysis when standard clinical indications are present, and ongoing monitoring for signs of rejection or infection. Persistent or unexplained dysfunction should prompt consideration of biopsy according to immunologic risk, local protocol, and the clinical trajectory. DGF may be common in high-risk deceased-donor transplantation, but it should remain a diagnosis reached after deliberate exclusion of reversible causes rather than a default explanation for poor early graft function.

Early immunosuppressive strategy may also influence the interpretation and management of poor early graft function. Calcineurin inhibitors, particularly tacrolimus, can cause dose-dependent afferent arteriolar vasoconstriction and may contribute to functional nephrotoxicity during a period when the allograft is already vulnerable to ischemia-reperfusion injury. In recipients at high risk for DGF, some centers consider delayed initiation or reduced early exposure to tacrolimus, especially when lymphocyte-depleting or interleukin-2 receptor antagonist induction provides immunologic cover. This strategy should not be applied uniformly, because under-immunosuppression may increase rejection risk, particularly in sensitized recipients. Therefore, tacrolimus timing and target trough levels should be individualized according to donor risk, early graft function, immunologic risk, and center-specific protocols.

Future work should also evaluate biomarkers that may detect early allograft injury before conventional clinical markers become definitive. Serum creatinine is a delayed and nonspecific marker of early graft injury, while urine output may be influenced by diuretics, volume status, and ischemia-reperfusion injury. Biomarkers such as neutrophil gelatinase-associated lipocalin (NGAL) and kidney injury molecule-1 (KIM-1) may provide earlier signals of tubular injury, whereas donor-derived cell-free DNA may offer a noninvasive marker of allograft injury with potential relevance to rejection, ischemic injury, and postoperative graft surveillance. At present, these biomarkers should be considered investigational or adjunctive in the immediate perioperative setting. Future prospective studies should define how biomarker-guided algorithms can distinguish DGF from rejection, vascular compromise, drug toxicity, and other reversible causes of early graft dysfunction.

ERAS Pathways as an Implementation Framework

ERAS pathways provide a practical method for translating evidence and multidisciplinary expertise into reliable perioperative processes. Kidney transplant ERAS recommendations include patient education, coordinated preoperative preparation, multimodal analgesia, early mobilization, nutrition when clinically appropriate, prevention of nausea and ileus, structured communication, and standardized escalation criteria [17]. Implementation studies suggest that ERAS is feasible in kidney transplantation and may improve recovery-related outcomes and length of stay without compromising graft-specific care [18-20].

For kidney transplantation, ERAS should not be interpreted as a fixed accelerated-discharge protocol. Graft function, hemodynamic stability, urinary drainage, electrolyte control, immunosuppressive monitoring, and infection risk must take precedence over arbitrary discharge targets. Instead, ERAS should be viewed as an implementation scaffold that standardizes the aspects of care most vulnerable to variation: preoperative checklists, medication reconciliation, fluid selection, blood pressure documentation, pain management, catheter-management plans, early mobilization, and criteria for imaging or surgical review.

**Figure 1:**
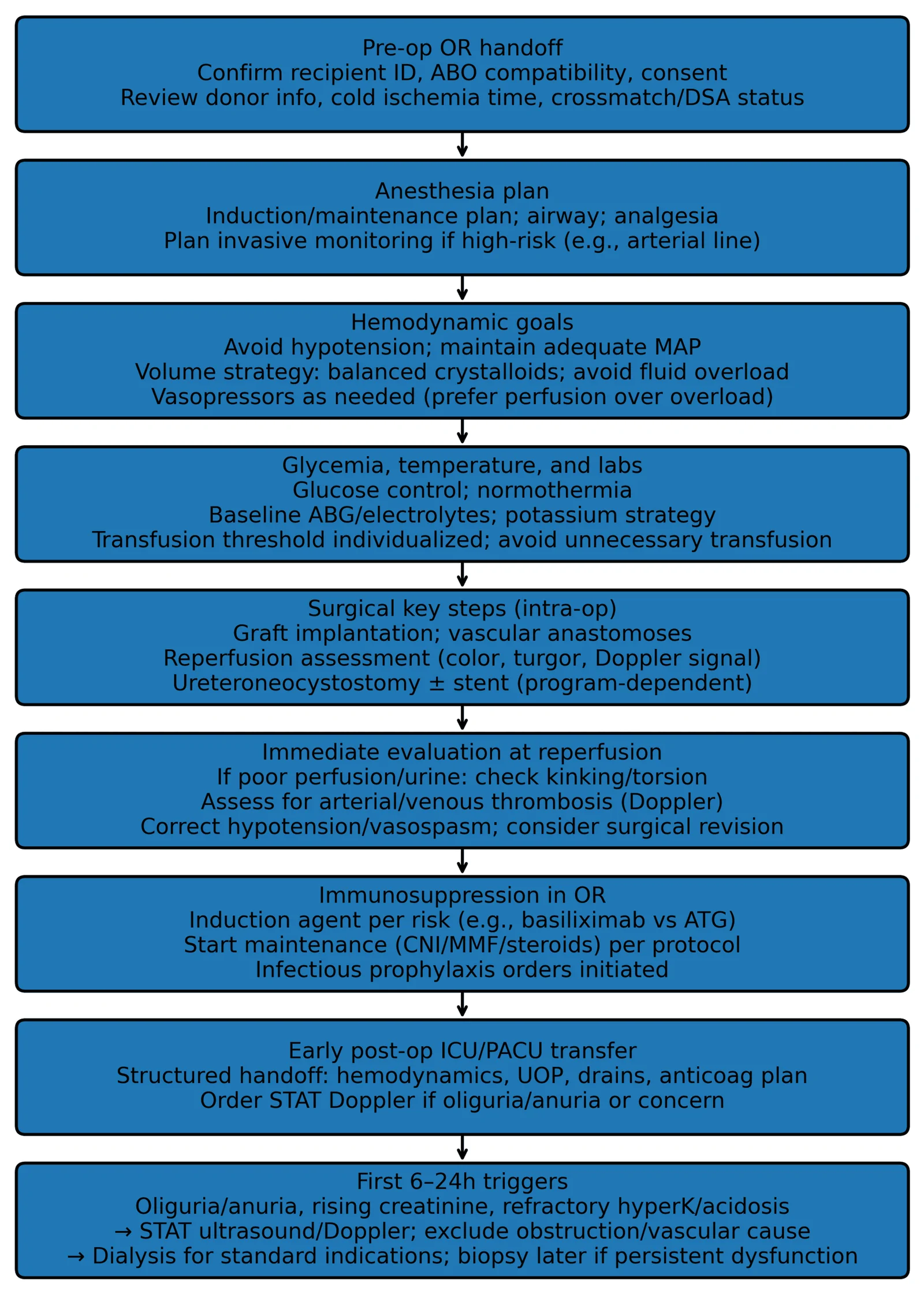
Peri-operative (during transplant) kidney transplant care pathway OR, operating room; DSA, donor-specific antibodies; MAP, mean arterial pressure; ATG, anti-thymocyte globulin; CNI, calcineurin inhibitor; MMF, mycophenolate mofetil; PACU, post-anesthesia care unit; UOP, urine output.

A transplant-specific ERAS pathway should include explicit triggers for escalation, including persistent oliguria despite hemodynamic optimization, increasing vasopressor requirement, new hyperkalemia, worsening acidosis, unexplained anemia, absent or deteriorating Doppler signals, and concern for urinary obstruction or leak. These elements are summarized in Table 2, while the proposed multidisciplinary perioperative workflow is illustrated in Figure 1.

**Table 2:** Peri-operative domains influencing early allograft function DSA, donor-specific antibodies; DGF, delayed graft function; RCT, randomized controlled trial; US, ultrasound.

Domain	Why it matters	Practical operational focus
Transplant-specific OR handoff	Prevents errors in immunologic/technical plan	Confirm ABO/crossmatch/DSA, ischemia times, last dialysis, induction plan
Hemodynamics	Hypotension worsens perfusion and may increase DGF risk	Perfusion-focused stability; minimize time in hypotension; early vasopressors when appropriate
Fluids	Overload worsens oxygenation and stability	Judicious volume; balanced crystalloids supported by RCT evidence
Electrolytes/acid–base	HyperK/acidosis destabilizes patient and perfusion	Planned monitoring and rapid treatment; dialysis readiness
Reperfusion troubleshooting	Technical causes are time-sensitive	Immediate assessment; low threshold for Doppler and revision if suspected
Handoff and escalation	Prevents “wait-and-see” delays	Structured transfer; explicit triggers for early Doppler/US

Limitations of the evidence

The evidence base for perioperative kidney transplant care remains uneven. The association between intraoperative hypotension and DGF is clinically compelling, but most data are observational and cannot establish a universal causal blood pressure threshold [15]. Similarly, vasopressor choice, optimal monitoring strategy, and ideal fluid volume remain incompletely defined and are influenced by recipient cardiovascular reserve, donor risk, surgical technique, and center-specific practice.

BEST-Fluids provides strong randomized evidence favoring balanced crystalloid over saline in deceased-donor transplantation; however, its findings should be interpreted within the context of the studied population, fluid protocol, and health-system setting [16]. Evidence regarding living-donor transplantation, high-risk cardiac recipients, intraoperative goal-directed hemodynamic algorithms, and specific vasopressor strategies remains more limited. Finally, DGF definitions based on dialysis within the first postoperative week may be influenced by center-specific dialysis practices, which complicate comparison between studies [7-10].

Discussion

Early kidney allograft function is determined by the interaction between baseline donor-recipient risk and the quality of perioperative care. Donor type, donor age, ischemia duration, graft preservation strategy, recipient cardiovascular reserve, immunologic risk, and surgical complexity establish the underlying probability of delayed graft recovery. However, these baseline factors do not negate the importance of potentially modifiable perioperative exposures. Hemodynamic instability, inappropriate fluid administration, metabolic derangements, excessive transfusion, delayed recognition of technical complications, and fragmented communication between teams may amplify ischemia-reperfusion injury and contribute to avoidable allograft dysfunction.

DGF remains a clinically important outcome because it is associated with prolonged hospitalization, increased healthcare utilization, more complex postoperative care, and adverse long-term graft outcomes in some recipient populations [7-11]. The conventional definition of DGF as dialysis requirement during the first postoperative week is practical and widely used; however, it has important limitations. The decision to initiate dialysis may vary between centers and clinicians according to local thresholds for hyperkalemia, acidosis, volume overload, and uremic symptoms. Therefore, DGF should be interpreted as a clinically useful outcome measure rather than a uniform biological entity. In practice, the central question is not simply whether dialysis is required, but whether poor early allograft function represents expected ischemia-reperfusion injury or an unrecognized reversible vascular, urological, or hemodynamic complication.

Donor and graft characteristics should guide the intensity of perioperative vigilance rather than create therapeutic nihilism. Kidneys from deceased donors, particularly those exposed to prolonged cold ischemia, donation after circulatory death, older donor age, or donor acute kidney injury, have a higher baseline susceptibility to ischemia-reperfusion injury and delayed recovery [7-12]. Risk-prediction models may help identify recipients at increased likelihood of DGF, but these models should be used to support planning, communication, and postoperative surveillance rather than to assume that poor graft function is unavoidable [12]. A recipient receiving a high-risk deceased-donor kidney may appropriately be expected to have delayed creatinine improvement; however, this expectation should not delay graft Doppler ultrasonography, surgical assessment, or investigation for obstruction when clinical findings are atypical.

The relationship between intraoperative blood pressure and early allograft function is one of the most clinically important areas of perioperative care. The transplanted kidney may have impaired autoregulatory capacity immediately after implantation because of ischemia-reperfusion injury, endothelial dysfunction, vasoplegia, and recipient vascular disease. Consequently, sustained hypotension may reduce graft perfusion and contribute to tubular injury. Observational evidence has demonstrated an association between intraoperative hypotension and DGF, supporting the principle that hypotension avoidance should be considered a central component of graft-protective care [15]. Nevertheless, the available evidence does not define a universal MAP threshold that applies to every recipient. Blood pressure goals should be individualized according to preexisting hypertension, cardiac function, baseline vascular disease, donor risk, vasopressor requirement, and the duration of hypotension.

From a practical intraoperative perspective, a MAP of at least 90 mmHg around graft reperfusion is a reasonable target when clinically tolerated, particularly in recipients with chronic hypertension, high DGF risk, or deceased-donor grafts exposed to prolonged ischemia. However, this target should not be pursued through indiscriminate fluid loading. Blood pressure goals should be individualized according to baseline recipient blood pressure, cardiac function, vasopressor requirement, donor and graft risk, and surgical conditions. The clinical priority is to minimize both the depth and duration of hypotension while maintaining adequate graft perfusion without causing cardiopulmonary overload.

This distinction is clinically relevant because treating every reduction in blood pressure with fluid alone may be harmful. Kidney transplant recipients frequently have limited cardiac reserve, left ventricular hypertrophy, pulmonary hypertension, or chronic volume expansion related to kidney failure. In such patients, excessive crystalloid administration may worsen pulmonary edema, impair oxygenation, increase right-sided cardiac pressures, and delay postoperative recovery. A more physiologically rational approach is to identify the dominant mechanism of hypotension. True intravascular depletion may require volume replacement, whereas vasodilatory hypotension may respond more appropriately to early vasopressor support. Hemorrhage, arrhythmia, impaired ventricular function, and technical graft-related complications should also be considered when hemodynamic instability persists despite initial treatment.

Fluid composition is another modifiable perioperative exposure. The BEST-Fluids randomized controlled trial provides the strongest available evidence that balanced crystalloid should be preferred over saline in deceased-donor kidney transplantation [16]. The trial demonstrated a lower incidence of DGF with Plasma-Lyte 148 than with saline, providing a directly actionable intervention that can be incorporated into routine perioperative protocols. The relevance of this finding extends beyond fluid selection alone. Balanced crystalloid use may reduce chloride exposure and hyperchloremic acidosis, thereby simplifying acid-base interpretation and reducing one potential contributor to physiological instability during a period of limited graft reserve. However, fluid type should not be confused with fluid quantity. Even balanced crystalloids can be harmful when given in excessive volumes to recipients with poor cardiac reserve or evolving pulmonary congestion.

Balanced crystalloids and normal saline differ in clinically relevant ways during kidney transplantation. Normal saline has a supraphysiologic chloride concentration and may contribute to hyperchloremic metabolic acidosis, which can complicate perioperative acid-base management and may adversely affect renal perfusion physiology. Balanced crystalloids contain lower chloride concentrations and a more physiologic electrolyte profile. In deceased-donor kidney transplantation, the BEST-Fluids randomized trial demonstrated a lower incidence of DGF with balanced crystalloid compared with saline, supporting balanced crystalloids as the preferred default perioperative crystalloid in this setting. Nevertheless, fluid type should not be separated from fluid volume: even balanced crystalloids can worsen pulmonary edema and cardiac strain if administered excessively. Therefore, current practice should favor balanced crystalloids over saline when appropriate, combined with individualized volume administration guided by hemodynamics, blood loss, oxygenation, cardiac reserve, and graft reperfusion status.

Perioperative metabolic management also deserves greater emphasis. Hyperkalemia, metabolic acidosis, anemia, hypocalcemia, hypomagnesemia, hypothermia, and hyperglycemia may compromise myocardial performance, predispose to arrhythmia, impair vascular tone, and complicate the interpretation of early graft dysfunction. These abnormalities may originate from the recipient’s baseline kidney failure, incomplete dialysis, transfusion, tissue injury, prolonged surgery, or reduced clearance before allograft function is established. A proactive strategy that includes preoperative assessment, repeated biochemical monitoring in high-risk cases, early treatment of severe hyperkalemia, temperature preservation, glucose management, and readiness to provide postoperative dialysis when clinically indicated may prevent secondary physiological insults during the vulnerable reperfusion period.

Reperfusion should be regarded as a diagnostic event rather than a passive waiting period for urine production. Graft color, turgor, arterial pulsatility, venous drainage, and immediate urine output can provide useful clues, but none alone reliably confirms adequate allograft perfusion. Persistent oliguria after reperfusion may occur with ischemia-reperfusion injury and does not necessarily indicate surgical failure. Conversely, early urine production does not exclude arterial thrombosis, venous congestion, kinking, torsion, anastomotic narrowing, graft malposition, or evolving ureteric obstruction. Therefore, abnormal reperfusion findings require parallel assessment of recipient hemodynamics and technical graft factors. Intraoperative Doppler ultrasonography can assist in evaluating arterial and venous patency when perfusion is uncertain, although it should complement direct surgical assessment rather than replace it [20].

The immediate postoperative period should be viewed as a continuation of the reperfusion assessment rather than a separate phase of care. DGF, vascular thrombosis, urinary obstruction, urine leak, hemodynamic injury, acute rejection, and medication-related nephrotoxicity may present with overlapping features, including oliguria, rising serum creatinine, hyperkalemia, metabolic acidosis, graft discomfort, or volume overload. Structured handoff from the operating room to the recovery area, intensive care unit, or transplant ward is therefore essential. The receiving team should understand the donor and graft risk profile, ischemia times, intraoperative hemodynamic exposure, fluid balance, blood loss, transfusion requirement, urine output after reperfusion, induction immunosuppression, anticoagulation plan, and any intraoperative concern regarding vascular or ureteric anatomy.

Early Doppler ultrasonography should be considered promptly when there is abrupt oliguria, persistent graft dysfunction that is disproportionate to anticipated ischemia time, graft tenderness, unexplained anemia, deteriorating hemodynamics, or concern for vascular compromise [13]. Urological complications should also remain within the differential diagnosis, particularly when hydronephrosis, pelvic discomfort, decreased urine output, high drain output, or an unexplained increase in creatinine is present [14]. The practical implication is that DGF should be a diagnosis of exclusion in the first postoperative period. Supportive management may be appropriate once vascular and urological causes have been reasonably excluded, but an assumption of “expected DGF” should never delay urgent imaging or surgical reassessment when the clinical trajectory is concerning.

The role of transfusion and anticoagulation illustrates the need for individualized decision-making. Kidney transplant recipients may have uremic platelet dysfunction, recent antiplatelet or anticoagulant exposure, vascular calcification, and technically difficult iliac vessel dissection, all of which increase the risk of bleeding. At the same time, arterial or venous thrombosis can result in irreversible graft loss. Transfusion should therefore be guided by active bleeding, hemodynamic instability, hemoglobin trajectory, myocardial ischemia risk, and oxygen-delivery requirements rather than an isolated hemoglobin value. Avoidable transfusion should be minimized when feasible, particularly in sensitized recipients, because blood exposure may contribute to HLA sensitization and future immunologic complexity [11]. However, concerns about sensitization must never delay transfusion when it is required to manage hemorrhage or maintain adequate tissue oxygen delivery.

ERAS pathways offer a practical mechanism for translating these concepts into reliable clinical processes. Their value in kidney transplantation is not limited to reducing length of stay or accelerating discharge. Rather, they can standardize areas of care that are especially vulnerable to variability, including preoperative assessment, medication reconciliation, patient education, fluid selection, multimodal analgesia, communication during handoff, early mobilization, catheter management, and explicit escalation criteria [17-20]. A kidney transplant ERAS pathway should preserve clinical flexibility because recovery is influenced by graft quality and DGF risk. It should therefore be considered an implementation framework rather than a rigid protocol.

A transplant-specific ERAS pathway should include predefined triggers for escalation, such as persistent oliguria despite optimization of hemodynamics, progressive hyperkalemia or acidosis, new vasopressor dependence, unexpected anemia, deteriorating Doppler signals, graft pain, or concern for urinary obstruction or leak. These triggers may reduce delays in recognizing complications that are surgically reversible or require urgent nephrology input. The proposed perioperative pathway in Figure 1 and the practical care bundle in Table 1 provide a structured framework for applying these principles from preoperative optimization through early graft surveillance.

Several limitations of the current evidence should be acknowledged. First, much of the evidence linking intraoperative hypotension to DGF is observational, and residual confounding is likely because hypotension may reflect underlying recipient illness, donor risk, blood loss, or surgical complexity rather than an isolated causal exposure [15]. Second, the optimal blood pressure target, vasopressor choice, fluid volume strategy, and intensity of invasive monitoring remain uncertain and are likely to differ according to recipient cardiac function, donor source, ischemia exposure, and local expertise. Third, DGF definitions vary in clinical interpretation because the use of dialysis may be influenced by center-specific practice. Finally, ERAS studies in kidney transplantation are still relatively limited, with variable pathway components and outcomes, making it difficult to determine which individual interventions provide the greatest benefit [17-20].

Future research should focus on pragmatic, multicenter studies that evaluate modifiable perioperative exposures using standardized definitions and clinically meaningful endpoints. Important priorities include cumulative duration below individualized perfusion thresholds, comparative vasopressor strategies, dynamic versus conventional approaches to fluid assessment, transfusion thresholds, standardized postoperative imaging algorithms, and the effect of structured escalation pathways on time to diagnosis of vascular and urological complications. Future studies should also distinguish graft dysfunction caused by ischemia-reperfusion injury from dysfunction related to potentially reversible surgical or hemodynamic events. Harmonized reporting of dialysis use, creatinine recovery, graft survival, rejection, length of stay, and resource utilization would strengthen the ability to compare protocols across transplant centers.

In summary, the most defensible contemporary approach to perioperative kidney transplant care is risk-adapted and multidisciplinary. It begins with optimization of recipient volume status, electrolytes, cardiovascular reserve, coagulation status, and immunologic planning; continues with perfusion-focused intraoperative management, balanced crystalloid use, avoidance of sustained hypotension, and early recognition of reperfusion abnormalities; and extends through structured postoperative surveillance for DGF and reversible graft complications. This strategy may not eliminate graft dysfunction caused by severe ischemia-reperfusion injury, but it can reduce avoidable secondary insults, improve communication across care transitions, and shorten the time to diagnosis of complications in which rapid intervention may preserve allograft function.

## Conclusions

Perioperative care is a major determinant of early kidney allograft function. While donor quality, ischemia time, and recipient comorbidity determine baseline DGF risk, modifiable factors such as hypotension, inappropriate fluid therapy, metabolic instability, and delayed recognition of technical complications can worsen early graft injury.

A risk-adapted approach should include preoperative optimization, clear communication of donor and recipient risk factors, avoidance of sustained hypotension, individualized fluid therapy with balanced crystalloids as the preferred option in deceased-donor transplantation, and prompt assessment of abnormal reperfusion. During the first postoperative days, DGF should be considered only after reversible vascular, urological, and hemodynamic causes of graft dysfunction have been excluded.

Structured handoff, early surveillance, timely Doppler imaging when indicated, and ERAS-informed multidisciplinary pathways may reduce avoidable delays and support early allograft recovery.

## References

[1] Kim H, Jung H (2024). Considerations regarding anesthesia for renal transplantation. Anesth Pain Med (Seoul).

[2] Tena B, Vendrell M (2020). Perioperative considerations for kidney and pancreas-kidney transplantation. Best Pract Res Clin Anaesthesiol.

[3] Goyal VK, Shekhrajka P, Mittal S (2025). Perioperative considerations in kidney transplantation: An anaesthesiologist's perspective. World J Transplant.

[4] Sicova M, McGinn R, Emerson S (2024). Association of intraoperative hypotension with delayed graft function following kidney transplant: A single centre retrospective cohort study. Clin Transplant.

[5] Collins MG, Fahim MA, Pascoe EM (2023). Balanced crystalloid solution versus saline in deceased donor kidney transplantation (BEST-Fluids): A pragmatic, double-blind, randomised, controlled trial. Lancet.

[6] Tan JH, Bhatia K, Sharma V, Swamy M, van Dellen D, Dhanda R, Khambalia H (2022). Enhanced recovery after surgery recommendations for renal transplantation: Guidelines. Br J Surg.

[7] Elsabbagh AM, Ghoneim I, Moiz A, Welch K, Brown JS (2022). Enhanced recovery after surgery pathway in kidney transplantation: The road less traveled. Transplant Direct.

[8] Scuffell C, Ashton K, Houston J (2022). Introducing an Enhanced Recovery After Surgery (ERAS) programme within the field of renal transplantation - The early Newcastle experience. Clin Nutr ESPEN.

[9] Romano F, Angelico R, Toti L (2025). The enhanced recovery after surgery pathway is safe, feasible and cost-effective in delayed graft function after kidney transplant. J Clin Med.

[10] Perico N, Cattaneo D, Sayegh MH, Remuzzi G (2004). Delayed graft function in kidney transplantation. Lancet.

[11] Siedlecki A, Irish W, Brennan DC (2011). Delayed graft function in the kidney transplant. Am J Transplant.

[12] Ojo AO, Wolfe RA, Held PJ, Port FK, Schmouder RL (1997). Delayed graft function: Risk factors and implications for renal allograft survival. Transplantation.

[13] Irish WD, Ilsley JN, Schnitzler MA, Feng S, Brennan DC (2010). A risk prediction model for delayed graft function in the current era of deceased donor renal transplantation. Am J Transplant.

[14] Jadlowiec CC, Hippen B, Gill J (2023). Current opinions on DGF management practices: A survey of the United States and Canada. Clin Transplant.

[15] Kim DW, Tsapepas D, King KL (2020). Financial impact of delayed graft function in kidney transplantation. Clin Transplant.

[16] Ponticelli C, Reggiani F, Moroni G (2022). Delayed graft function in kidney transplant: Risk factors, consequences and prevention strategies. J Pers Med.

[17] Ahlmark A, Sallinen V, Eerola V, Lempinen M, Helanterä I (2024). Characteristics of delayed graft function and long-term outcomes after kidney transplantation from brain-dead donors: A single-center and multicenter registry-based retrospective study. Transpl Int.

[18] Buta BJ, Walston JD, Godino JG (2016). Frailty assessment instruments: Systematic characterization of the uses and contexts of highly-cited instruments. Ageing Res Rev.

[19] Guralnik JM, Simonsick EM, Ferrucci L (1994). A short physical performance battery assessing lower extremity function: Association with self-reported disability and prediction of mortality and nursing home admission. J Gerontol.

[20] Pavasini R, Guralnik J, Brown JC (2016). Short Physical Performance Battery and all-cause mortality: Systematic review and meta-analysis. BMC Med.

